# Association between CTLA-4 60G/A and -1661A/G Polymorphisms and the Risk of Cancers: A Meta-Analysis

**DOI:** 10.1371/journal.pone.0083710

**Published:** 2013-12-23

**Authors:** Qing Yan, Pin Chen, Ailin Lu, Peng Zhao, Aihua Gu

**Affiliations:** 1 Department of neurosurgery, the First Affiliated Hospital, Nanjing Medical University, Nanjing, China; 2 Key Laboratory of Modern Toxicology of Ministry of Education, School of Public Health, Nanjing Medical University, Nanjing, China; MOE Key Laboratory of Environment and Health, School of Public Health, Tongji Medical College, Huazhong University of Science and Technology, China

## Abstract

**Purpose:**

CTLA-4 is one of the most fundamental immunosuppressive cotykines which belongs to the immunoglobulin super-family, and is expressed mainly on activated T cells. Previous studies have reported the existence of CTLA4 60G/A and CTLA4 -1661A/G polymorphism in cancers. However, the effects remain conflicting. Hence, we performed a meta-analysis to investigate the association between these polymorphisms and cancer risk.

**Methods:**

We searched the Pubmed and Web of Science databases until October 24, 2013 to obtain relevant published studies. Pooled odds ratios (ORs) and corresponding 95% confidence intervals (CIs) for the relationship between CTLA4 gene polymorphisms and cancer susceptibility were calculated by stata 11 software. Heterogeneity tests, sensitivity analyses and publication bias assessments were also performed in our meta-analysis.

**Results:**

A total of 22 articles comprising 31 case-control studies concerning the CTLA-4 60G/A and CTLA-4 -1661A/G polymorphisms were included in the meta-analysis. The pooled results suggested the CTLA-4 60G/A polymorphism was significantly associated with an increased skin cancer risk (AA vs. GG: OR = 1.32, 95%CI = 1.09-1.59; AA vs. GA+GG: OR = 1.26, 95%CI = 1.07-1.48). For CTLA-4 -1661 A/G polymorphism, the results showed that the CTLA-4 -1661A/G polymorphism was significantly associated with an increased cancer risk (GA vs. AA: OR = 1.44, 95%CI = 1.13–1.82; GA+GG vs. AA: OR = 1.35, 95%CI = 1.07–1.69; G vs. A: OR = 1.21, 95%CI = 1.01–1.47), especially in gastric cancer, breast cancer, other cancers and in Asians population subgroups.

**Conclusion:**

Our meta-analysis suggests that the CTLA-4 -1661A/G polymorphism is a potential factor for the susceptibility of cancer, especially in gastric cancer, breast cancer and other cancers, and the CTLA-4 60G/A polymorphism is significantly associated with increased skin cancer risk. The effect of the CTLA-4 -1661A/G polymorphism on cancer susceptibility especially exists in Asians and population based subjects.

## Introduction

Cancer is a major cause of death in most countries, and it is estimated that the number of new cases of patients will be more than 15 million in the coming decade, creating a substantial worldwide public health burden [1]. Recently researches have shown that cancer is the result of complex interactions in many factors, especially between inherited and environmental factors [2]. However, the exact aetiology and mechanism of carcinogenesis still have not been clearly elucidated. In recently years, it has been velar that genetic variation is an important factor contributes to the development and progression of cancer, and an increasing number of studies have focused on the interactions between genetic factors and cancer susceptibility [3].

Cytotoxic T lymphocyte antigen-4 (CTLA-4), one of the most fundamental immunosuppressive cotykines, is a co-inhibitory molecule belonging to the immunoglobulin super-family, and is expressed mainly on activated T cells [4]. This molecule is a homodimeric glycoprotein receptor on CTLs and CD28 homologue, although CTLA-4 shares homology with CD28, it has a higher binding affinity of the CD80/CD86 ligands than CD28, which results in the inhibition of T-cell proliferation, activation and cytokine production [5], [6]. Recent studies showed that mice deficient the CTLA-4 gene were born healthy but died early due to severe lymphoproliferative disorders and autoimmune diseases [7]. In tumor, CTLA-4 is upregulated on the T cells with the help of TGF-β (a suppressive cytokine secreted by the tumor cells),and during the early stage of tumorigenesis, CTLA-4 may elevate the T-cell activation threshold, thereby attenuating the antitumor response and increasing cancer susceptibility [8]. CTLA-4 protein is encoded by CTLA-4 gene, which is located in several immune regulatory genes area of human chromosome 2(2q33–2q37). More than 100 single nucleotide polymorphisms (SNPs) have been identified in the CLTA-4 gene. Among the CTLA-4 gene polymorphisms, two polymorphisms including CTLA4 60G/A (rs3087243) in the 3’-UTR, and CTLA4 -1661A/G (rs4553808) in promoter region were widely studied and have been reported to be associated with susceptibility to inflammatory diseases, autoimmune diseases and cancers [9], [10].

In recent years, CTLA-4 gene has been the research focuses in the scientific community, and a number of epidemiological studies have been performed to assess the possible interaction between the CTLA-4 gene polymorphism and cancer susceptibility, including breast cancer, cervical cancer, lung cancer, glioma and so on. However, the results of the different studies are conflicting. For example, Hou et al. found that CTLA-4 -1661A/G is associated with significantly increased risk of gastric cancer, but Hadinia et al. reported that no significant association was found between CTLA-4 -1661A/G polymorphism and colorectal cancer [11], [12]. Thus, the association between CTLA-4 gene polymorphisms and cancer susceptibility requires further investigation. Hence, in this paper, we perform a meta-analysis on previous reports to investigate the association of CTLA-4 gene polymorphism with cancer.

## Materials and Methods

### Study eligibility and validity assessment

We performed a computerized literature search of the Pubmed and Web of Science databases using the search terms “CTLA-4 or Cytotoxic T lymphocyte antigen-4” and “polymorphism” updated until October 24, 2013. To obtain all eligible publications, the related reference articles were reviewed to identify other potentially eligible publications. The studies not matching the eligible criteria were excluded in our meta-analysis.

### Inclusion criteria

All studies were included in the meta-analysis if met the following criteria: 1) articles on CTLA4 60G/A (rs3087243) and/or CTLA4 -1661A/G (rs4553808) and cancer risk; 2) use a human case-control design; 3) contain sufficient published data for estimation of odd ratios (ORs) with a 95% confidence interval (CI).

### Data extraction

According to the inclusion criteria listed above, necessary data from all of the eligible publications were extracted by two investigators (Yan and Wang) independently. Disagreement between the two investigators were resolved by discussion until a consensus was reached. For each study, the following information was extracted including: the first author’s name, publication data, country of origin, genotyping methods, ethnicities of the sample population, cancer type, source of control group, total number of cases and controls, and the number of cases and controls with CTLA-4 gene polymorphisms.

### Statistical methods

First, we assessed HWE for the controls in each study. The strength of the association between variant allele of CTLA-4 gene polymorphisms and cancer risk was assessed by ORs with 95% confidence intervals (CIs). The statistical significance of the pooled OR was calculated by the Z test, a P<0.05 was considered to be statistically significant (P-values were two sided). Analysis between homozygote model, heterozygote model, dominant model, recessive model and allelic models was also done to estimate cancer risk. Subgroup analyses were also conducted by HWE, cancer type (if a cancer type with only one individual study was combined into other cancer group), source of controls and ethnicity. Statistical heterogeneity was considered to be significant when the P was <0.05. In case of a significant heterogeneity, the pooled ORs were analyzed using a random effects model (the Dersimonian and Laird method) [13]. If insignificance (P>0.05) was found, a fixed-effects model (the Mantel-Haensze method) should be used [14]. The inter-study variance I^2^ (I^2^ = 100%×(Q–df)/Q) was used to quantitatively estimate heterogeneity, and the percentage of I^2^ was used to describe the extend of heterogeneity, where I^2^<25%,25–75%, and >75% represent low, moderate and high inconsistency, respectively[15], [16]. Additionally, sensitivity analyses were also performed by omitting each study to reflect the influence of individual data on summary ORs. Finally, publication bias was weighted by Begg’s funnel plot and Egger’ linear regression method, when P<0.05 was considered statistically significant [17]. All analyses were conducted by the software Stata (Version 11; Stata corporation, College Station, Texas, USA). All p-value were two-sided and a P<0.05 was considered to be statistically significant.

## Results

### Characteristics of included studies

**Figure 1:**
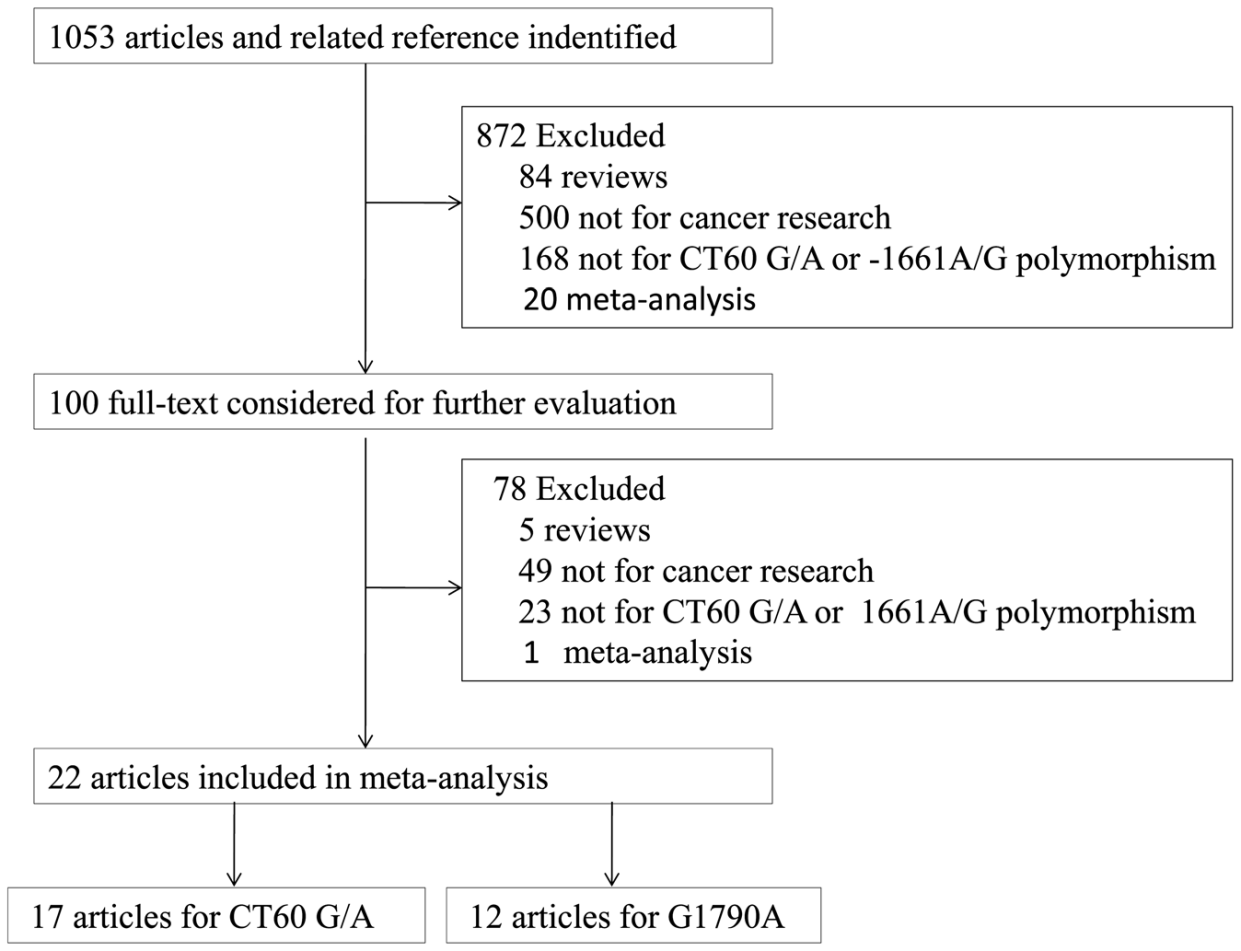
Study flow-chart illustrating the literature search and eligible study selection process.

The flow diagram illustrates the main reasons for studies searching and selecting (Figure 1), and the selected study characteristics were summarized in Table 1. A total of 22 articles comprising 31 case-control studies concerning the CTLA-4 60G/A (rs3087243) and/or CTLA-4 -1661A/G (rs4553808) polymorphisms were included in the meta-analysis.

**Table 1:** Characteristics of studies included in the meta-analysis.

First					Gene	Source of									
author	Year	Country	Ethnicity	Cancer type	type	controls	Cases	Controls		Case			Control		HWE
									MM	MW	WW	MM	MW	WW	
Chuang	2005	Germany	Caucasian	Thymoma	60 G/A	PB	125	173	40	61	24	43	95	35	Y
Cheng	2006	Taiwan	Asian	Gastric	60 G/A	HB	62	250	3	20	39	17	79	154	Y
Cozar	2007	Spain	Caucasian	Renal	60 G/A	PB	127	175	23	55	49	47	88	40	Y
Cozar	2007	Spain	Caucasian	Colorectal	60 G/A	PB	95	175	19	56	20	47	88	40	Y
Wang	2007	China	Asian	Breast	60 G/A	PB	117	148	24	47	46	18	56	74	Y
					–1661A/G	PB	109	148	62	45	2	111	35	2	Y
Hadinia	2007	Iran	Caucasian	Gastric	–1661A/G	PB	46	188	37	9	0	145	36	7	N
Hadinia	2007	Iran	Caucasian	Colorectal	–1661A/G	PB	109	188	74	33	2	145	36	7	N
Li	2008	China	Asian	Breast	60 G/A	PB	328	327	32	124	172	20	114	193	Y
Welsh	2009	USA	Caucasian	Skin	60 G/A	HB	1591	821	450	791	350	280	385	156	Y
Bouwhuis	2010	Netherland	Caucasian	Melanoma	60 G/A	PB	763	733	246	355	162	223	388	122	N
Hou	2010	China	Asian	Gastric	–1661A/G	PB	205	262	112	71	22	163	54	45	N
Kammerer	2010	Germany	Caucasian	Oral	–1661A/G	HB	40	83	35	4	1	48	25	10	Y
Rahimifar	2010	Iran	Caucasian	Cervical	–1661A/G	PB	55	110	25	28	2	74	31	5	Y
Khaghanzadeh	2010	Iran	Caucasian	Lung	60 G/A	PB	124	95	30	51	43	21	36	38	N
					–1661A/G	PB	126	118	87	36	3	91	23	4	Y
Liu	2011	China	Asian	Osteosarcoma	60 G/A	HB	267	282	176	77	14	188	83	11	Y
					–1661A/G	HB	267	282	177	76	14	197	73	12	Y
Cheng	2011	China	Asian	Esophageal	–1661A/G	PB	205	205	115	82	8	145	53	7	Y
Karabon	2011	Poland	Caucasian	Lung	60 G/A	HB	208	325	70	109	29	112	156	57	Y
Karabon	2012	Poland	Caucasian	Myeloma	60 G/A	PB	193	374	81	88	24	128	180	66	Y
Li	2012	China	Asian	Breast	60 G/A	PB	581	566	361	197	23	361	182	23	Y
					–1661A/G	PB	574	551	405	153	16	425	115	11	Y
Erfani	2012	Iran	Caucasian	HNSCC	60 G/A	HB	80	81	21	44	15	14	34	33	Y
Bharti	2013	India	Asian	Oral	60 G/A	PB	130	180	12	47	71	34	79	67	Y
					–1661A/G	PB	120	180	94	26	0	162	18	0	Y
Khorshied	2013	Egypt	Caucasian	Lymphoma	60 G/A	PB	181	200	36	94	51	44	96	60	Y
Liu	2013	China	Asian	Lymphoma	60 G/A	HB	291	300	197	84	10	208	82	10	Y
					–1661A/G	HB	291	300	220	66	5	216	78	6	Y
Feng	2013	China	Asian	Sarcoma	60 G/A	HB	308	362	210	87	11	243	105	14	Y
					–1661A/G	HB	308	362	209	83	16	252	96	14	Y
Total:					60 G/A		5571	5567	2031	2387	1153	2048	2326	1193	
					–1661A/G		2455	2977	1652	712	91	2174	673	130	

W: wide type alleles (60 G or –1661A); M: mutant type alleles (60 A or –1661G); HWE: Hardy-Weinberg Equilibrium; PB: population based; HB: hospital based; HNSCC: head and neck squamous cell carcinoma.

For CTLA-4 60G/A (rs3087243) polymorphism, there were 17 articles [18], [19], [20], [21], [22], [23], [24], [25], [26], [27], [28], [29], [30], [31], [32], [33], [34] met the inclusion criteria with 5571 cases and 5567 controls, 1 article (Cozar et al.) [20] provided 2 kinds of cancers (renal cancer and colorectal cancer) in CTLA-4 60G/A polymorphism, thus, each type of cancer in these articles was treated as a separated case-control study. So, there were a total of 18 case-control studies included in our meta-analysis. Among the 18 studies, there were 11 studies of population-based population, and 7 studies of hospital-based population. 18 studies included 8 studies of Asians and 10 studies of Caucasians. In the eligible studies, there were 3 studies of breast cancer, 2 studies of skin cancer, 2 studies of lung cancer, 2 studies of lymphoma, 2 studies of bone cancer, 1study of thymoma, 1 study of renal cancer, 1 study of multiple myeloma, 1 study of head and neck cancer, 1 study of gastric cancer, 1 study of colon carcinoma and 1 study of oral cancer. The distributions of the genotypes in the control groups in 2 studies were not in HWE [24], [25]. For CTLA-4 -1661A/G (rs4553808) polymorphism**,** 12 articles [11], [12], [21], [25], [26], [29], [31], [32], [34], [35], [36], [37] containing 13 individual case-control studies with 2455 cases and 2977 controls were included in our meta-analysis. 8 studies were carried out in Asian population and 5 studies were carried out in Caucasians. Among the eligible studies, there were 2 studies of gastric cancer, 2 studies of breast cancer, 2 studies of oral cancer, 2 studies of bone cancer, 1 study of lung cancer, 1 study of colorectal cancer, 1 study of cervical cancer, 1study of lymphoma and 1study of esophageal cancer. The control sources were population-based in 9 studies and hospital-based in 4 studies. The distributions of the genotypes in the control groups in 2 studies were not in HWE [11], [12].

### Main results of meta-analysis

**Figure 2:**
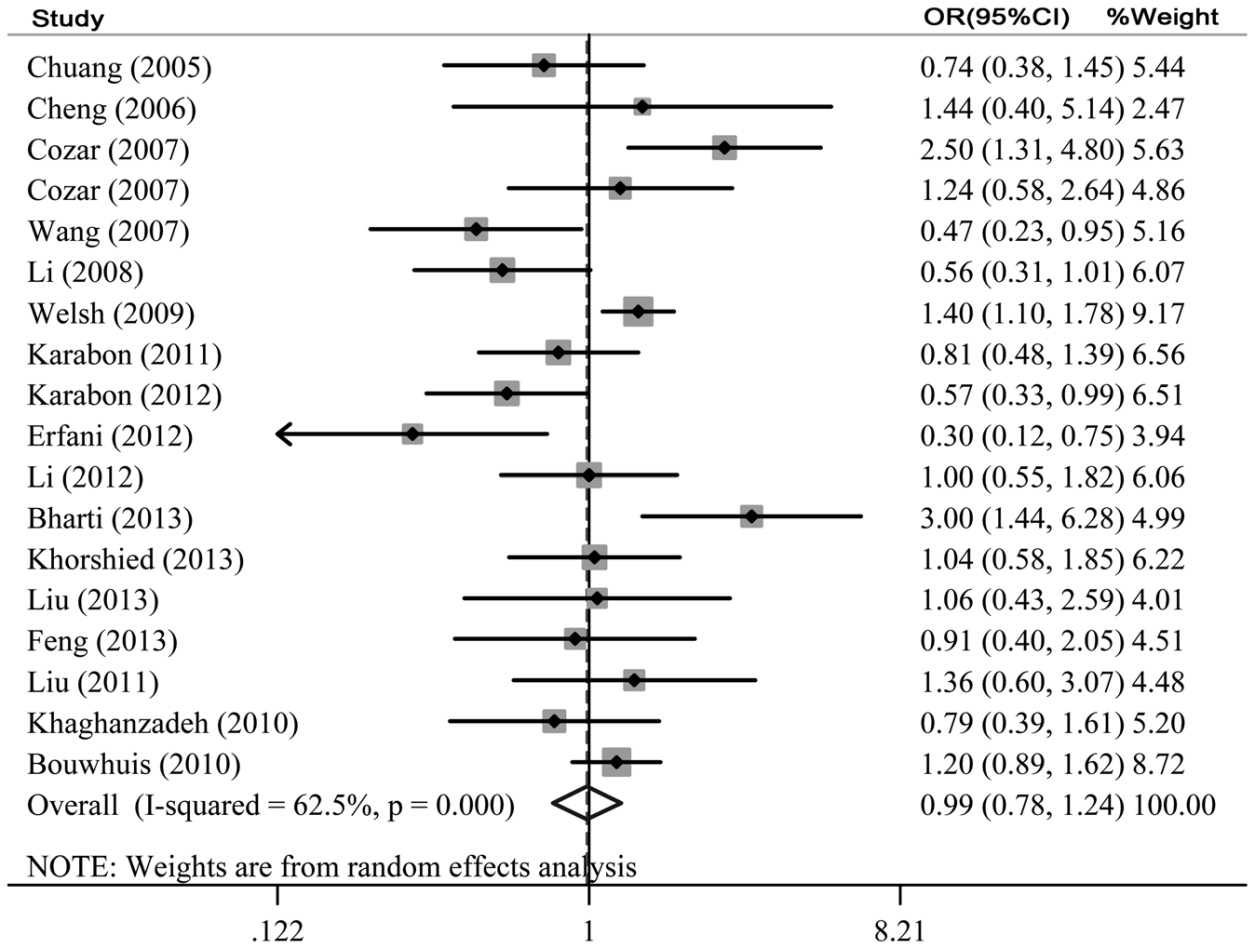
Forest plot of the association between cancer risk and the CTLA-4 60G/A polymorphism using the homozygote model (AA vs. GG).

The main results of meta-analysis about CTLA-4 60G/A polymorphism were shown in Table 2. Firstly, we conducted meta-analysis of the effect of CTLA-4 60G/A polymorphism on the susceptibility of cancers based on 18 case-control studies (Table 2, Figure 2). The results showed no significant association between the two in all five models (AA vs. GG: OR = 0.99, 95%CI = 0.78–1.24; GA vs. AA: OR = 1.03, 95%CI = 0.94–1.13; AA+AG vs. GG: OR = 1.01, 95%CI = 0.88–1.15; AA vs. GA+GG: OR = 0.98, 95%CI = 0.81–1.18; A vs. G: OR = 0.99, 95%CI = 0.89–1.11). We then performed the subgroup analyses stratified by cancer types, ethnicity and source of controls. The pooled ORs for homozygote model comparison and recessive model comparison suggested the CTLA-4 60G/A polymorphism was significantly associated with an increased skin cancer risk (AA vs. GG: OR = 1.32, 95%CI = 1.09–1.59; AA vs. GA+GG: OR = 1.26, 95%CI = 1.07–1.48). In the subgroup analysis by source of controls, we found that subjects with AA or AG genotype had 1.13 fold higher risk than those with GG genotype in hospital based population (AA+AG vs. GG: OR = 1.13, 95%CI = 1.01–1.28). The remaining subgroup pooled ORs from this analysis were insignificant (all P>0.05) (Table 3). Sensitivity analysis was then performed by excluding the studies with controls not in HWE. The results were similar to those when the studies with controls not in HWE were included (Table 2).

**Table 2:** Meta-analysis of the CTLA-4 60G/A (rs3087243) polymorphism and cancer risk.

		AA vs. GG				GA vs. GG				AA+AG vs. GG				AA vs. GA+GG				A vs. G			
	*Study*	*I^2^*	*Phet*	*OR*	*95% CI*	*I^2^*	*Phet*	*OR*	*95% CI*	*I^2^*	*Phet*	*OR*	*95% CI*	*I^2^*	*Phet*	*OR*	*95% CI*	*I^2^*	*Phet*	*OR*	*95% CI*
Overall	18	63	<0.001	0.99	0.78–1.24^*^	20	0.21	1.03	0.94–1.13	49	0.01	1.01	0.88–1.15^*^	63	<0.001	0.98	0.81–1.18^*^	68	<0.001	0.99	0.89–1.11^*^
Overall in HWE	16	66	<0.001	0.98	0.75–1.28^*^	13	0.30	1.07	0.97–1.19	52	0.01	1.02	0.88–1.19^*^	63	<0.001	0.97	0.79–1.18^*^	71	<0.001	0.99	0.88–1.13^*^
Breast	3	35	0.21	0.66	0.46–0.95	41	0.18	0.97	0.78–1.21	65	0.04	0.76	0.46–1.24^*^	0	0.58	0.77	0.60–0.97	69	0.04	0.83	0.63–1.09^*^
Skin	2	**0**	**0.45**	**1.32**	**1.09**–**1.59**	87	0.01	1.04	0.68–1.58^*^	84	0.01	1.10	0.78–1.57^*^	**0**	**0.50**	**1.26**	**1.07**–**1.48**	39	0.20	0.88	0.76–2.02
Lung	2	0	0.95	0.81	0.53–1.24	0	0.77	1.09	0.78–1.53	0	0.68	0.99	0.73–1.37	0	0.91	0.78	0.54–1.12	0	0.69	0.92	0.74–1.13
Lymphoma	2	0	0.98	1.04	0.64–1.70	0	0.76	1.12	0.83–1.51	0	0.87	1.10	0.83–1.46	0	0.81	0.94	0.63–1.39	0	0.80	1.03	0.84–1.27
Bone	2	0	0.49	1.11	0.63–1.97	0	0.90	0.97	0.76–1.25	0	0.74	0.99	0.78–1.26	0	0.50	1.12	0.64–1.97	0	0.59	1.01	0.82–1.24
Other	7	79	<0.001	1.08	0.59–1.99^*^	29	0.20	0.99	0.79–1.24	65	0.01	1.09	0.74–1.61^*^	79	<0.001	1.01	0.64–1.59^*^	82	<0.001	1.04	0.76–1.42^*^
Asian	8	60	0.01	1.01	0.66–1.55^*^	0	0.56	1.02	0.88–1.18	44	0.84	1.02	0.89–1.17	55	0.03	1.02	0.76–1.37^*^	69	0.002	1.00	0.91–1.11^*^
Caucasian	10	67	0.001	0.98	0.74–1.30^*^	42	0.80	1.04	0.93–1.17	56	0.02	1.01	0.84–1.22^*^	68	0.001	0.96	0.75–1.22^*^	70	<0.001	1.05	0.98–1.13^*^
Caucasian in HWE	8	73	0.001	0.95	0.65–1.39^*^	32	0.17	1.13	0.99–1.29	60	0.01	1.04	0.82–1.32^*^	71	0.001	0.91	0.67–1.24^*^	76	<0.001	0.98	0.81–1.19^*^
PB	11	68	0.001	0.99	0.73–1.36^*^	27	0.19	0.94	0.83–1.07	55	0.01	0.97	0.79–1.19^*^	69	<0.001	1.02	0.80–1.30^*^	73	<0.001	1.00	0.93–1.08^*^
PB in HWE	9	73	<0.001	0.99	0.66–1.49^*^	34	0.15	0.99	0.85–1.16	64	0.01	0.99	0.76–1.30^*^	70	0.001	1.01	0.75–1.35^*^	78	<0.001	1.01	0.82–1.24^*^
HB	7	54	0.04	0.98	0.67–1.43^*^	0	0.74	1.14	0.99–1.29	16	0.31	1.13	1.01–1.28	56	0.04	0.91	0.66–1.25^*^	58	0.03	1.08	0.99–1.17^*^

HWE: Hardy-Weinberg Equilibrium; PB: population based; HB: hospital based; Phet: P value for heterogeneity. ^*^Random-effects model was used when P value for heterogeneity test <0.05; otherwise, fixed-effects model was used.

**Table 3:** Meta-analysis of the CTLA-4 -1661A/G (rs4553808) polymorphism and cancer risk.

		GG vs. AA				GA vs. AA				GA+GG vs. AA				GG vs. GA+AA				G vs. A			
	*Study*	*I^2^*	*Phet*	*OR*	*95% CI*	*I^2^*	*Phet*	*OR*	*95% CI*	*I^2^*	*Phet*	*OR*	*95% CI*	*I^2^*	*Phet*	*OR*	*95% CI*	*I^2^*	*Phet*	*OR*	*95% CI*
Overall	13	0	0.57	0.96	0.72–1.28	**68**	**<0.001**	**1.44**	**1.13**–1.82^*^	**68**	**<0.001**	**1.35**	**1.07**–1.69^*^	0	0.56	0.86	0.65–1.15	**66**	**<0.001**	**1.21**	**1.01**–1.47^*^
Overall in HWE	10	0	0.68	1.15	0.81–1.63	**73**	**<0.001**	**1.40**	**1.05**–1.86^*^	**75**	**<0.001**	**1.36**	**1.03**–1.81^*^	0	0.83	1.09	0.77–1.53	72	<0.001	1.26	0.99–1.60^*^
Gastric	2	0	0.50	0.67	0.39–1.16	**51**	**0.15**	**1.65**	**1.14**–**2.40**	22	0.26	1.25	0.89–1.74	0	0.59	0.56	0.33–1.95	0	0.36	0.98	0.75–1.28
Breast	2	0	0.88	1.56	0.75–3.22	**62**	**0.11**	**1.55**	**1.21**–**1.98**	**60**	**0.11**	**1.55**	**1.22**–**1.97**	0	0.98	1.40	0.68–2.89	**41**	**0.19**	**1.45**	**1.18**–**1.80**
Oral	2	-	-	-	-	93	<0.001	0.77	0.07–8.57^*^	94	<0.001	0.72	0.06–8.92^*^	-	-	-	-	95	<0.001	0.73	0.07–7.56^*^
Bone	2	0	0.92	1.34	0.80–2.31	0	0.69	1.09	0.85–1.41	0	0.74	1.13	0.88–1.44	0	0.87	1.31	0.76–2.24	0	0.82	1.14	0.92–1.40
Other	5	0	0.88	0.96	0.53–1.74	**71**	**0.01**	**1.60**	**1.05**–2.46^*^	**69**	**0.01**	**1.51**	**1.02**–2.24^*^	0	0.93	0.83	0.46–1.49	**56**	**0.06**	**1.26**	**1.05**–**1.52**
Other in HWE	4	0	0.88	1.06	0.56–2.03	78	0.004	1.57	0.93–2.67^*^	76	0.01	1.13	0.91–2.47^*^	0	0.95	0.91	0.48–1.73	67	0.03	1.31	0.91–1.88^*^
Asian	8	0	0.65	1.1	0.80–1.50	**68**	**0.003**	**1.46**	**1.13**–1.88^*^	**63**	**0.01**	**1.39**	**1.11**–1.74^*^	0	0.45	0.97	0.72–1.32	**59**	**0.02**	**1.27**	**1.06**–1.53^*^
Asian in HWE	7	0	0.97	1.34	0.92–1.96	**69**	**0.004**	**1.41**	**1.07**–1.85^*^	**68**	**0.01**	**1.41**	**1.08**–1.83^*^	0	0.99	1.26	0.86–1.83	**61**	**0.02**	**1.32**	**1.07**–1.63^*^
Caucasian	5	0	0.55	0.48	0.22–1.04	74	0.004	1.26	0.67–2.35^*^	79	0.001	1.09	0.56–2.11^*^	0	0.81	0.45	0.21–1.00	78	0.001	0.95	0.53–1.68^*^
Caucasian in HWE	3	28	0.25	0.5	0.19–1.28	86	0.001	1.09	0.34–3.53^*^	88	<0.001	0.97	0.28–3.30^*^	0	0.51	0.48	0.19–1.25	88	<0.001	0.86	0.31–2.40^*^
PB	9	0	0.69	0.94	0.65–1.35	**7**	**0.38**	**1.74**	**1.49**–**2.04**	**20**	**0.27**	**1.6**	**1.38**–**1.86**	0	0.64	0.78	0.55–1.11	**40**	**0.40**	**1.37**	**1.20**–**1.56**
PB in HWE	6	0	0.95	1.37	0.81–2.31	**22**	**0.27**	**1.76**	**1.46**–**2.11**	**17**	**0.31**	**1.72**	**1.44**–**2.06**	0	0.92	1.16	0.69–1.94	**0**	**0.51**	**1.54**	**1.32**–**1.80**
HB	4	36	0.20	1.01	0.63–1.60	63	0.04	0.88	0.60–1.28^*^	74	0.01	0.84	0.55–1.29^*^	12	0.13	1.03	0.65–1.64	78	0.004	0.85	0.57–1.27^*^

HWE: Hardy-Weinberg Equilibrium; PB: population based; HB: hospital based; Phet: P value for heterogeneity. ^*^Random-effects model was used when P value for heterogeneity test <0.05; otherwise, fixed-effects model was used.

**Figure 3:**
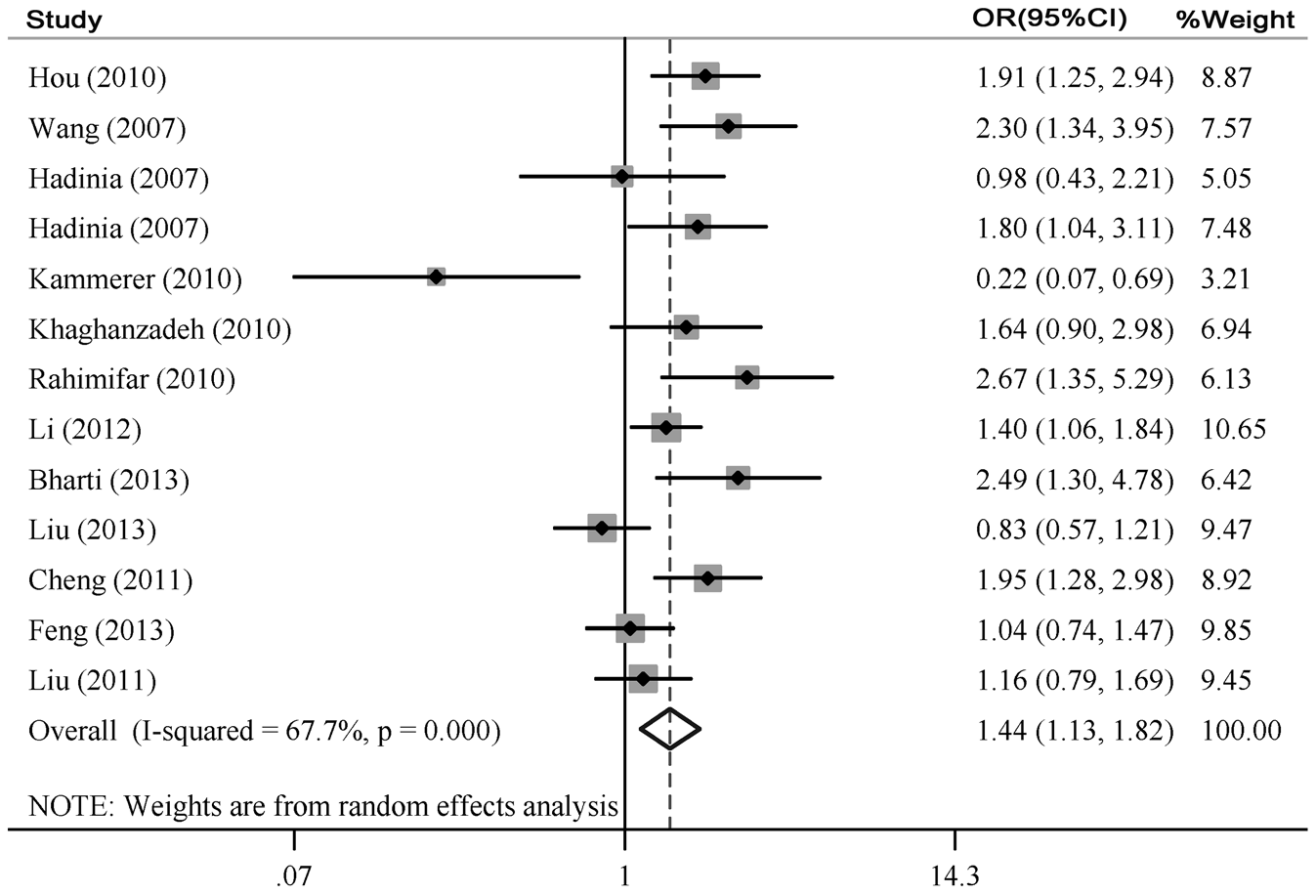
Overall association between the CTLA-4 -1661A/G polymorphism and cancer risk for all subjects using the heterozygote model (GA vs. AA).

The main results of meta-analysis about CTLA-4 -1661A/G polymorphism were shown in Table 3. The results on all 13 studies showed that the CTLA-4 -1661A/G polymorphism was significantly associated with an increased cancer risk (GA vs. AA: OR = 1.44, 95%CI = 1.13–1.82; GA+GG vs. AA: OR = 1.35, 95%CI = 1.07–1.69; G vs. A: OR = 1.21, 95%CI = 1.01–1.47) (Table 3, Figure 3). Subsequently, we performed subgroup analyses based on the difference of cancer type, ethnicity and source of controls. Significant associations were found in gastric cancer (GA vs. AA: OR = 1.65, 95%CI = 1.14–2.04), breast cancer (GA vs. AA: OR = 1.55, 95%CI = 1.21–1.98; GA+GG vs. AA: OR = 1.55, 95%CI = 1.22–1.97; G vs. A: OR = 1.45, 95%CI = 1.18–1.80) and other cancers (GA vs. AA: OR = 1.60, 95%CI = 1.05–2.46; GA+GG vs. AA: OR = 1.51, 95%CI = 1.02–2.24; G vs. A: OR = 1.26, 95%CI = 1.05–1.52). In the subgroup analysis by ethnicity, the significant association was found between the increased cancer risk and Asians (GA vs. AA: OR = 1.46, 95%CI = 1.13–1.88; GA+GG vs. AA: OR = 1.39, 95%CI = 1.11–1.74; G vs. A: OR = 1.27, 95%CI = 1.06–1.53). A marginal significant association between the CTLA-4 -1661A/G polymorphism and increased cancer risk was detected in population based group under heterozygote model, dominant model and allele model (GA vs. AA: OR = 1.74, 95%CI = 1.49–2.04; GA+GG vs. AA: OR = 1.60, 95%CI = 1.38–1.86; G vs. A: OR = 1.37, 95%CI = 1.20–1.56).The remaining pooled ORs from this meta-analysis were not significant (P>0.05) (Table 3). Then we performed reanalysis after exclusion the studies with controls not in HWE. The results from the heterozygote model comparison, dominant model comparison and allelic frequency comparison showed no evidence that the CTLA-4 -1661A/G polymorphism was significantly associated with an increased other cancers risk (GA vs. AA: OR = 1.57, 95%CI = 0.93–2.67; GA+GG vs. AA: OR = 1.13, 95%CI = 0.91–2.47; G vs. A: OR = 1.31, 95%CI = 0.91–1.88). The other results were similar to those when the studies with controls not in HWE were included (Table 3).

### Heterogeneity, sensitivity and publication bias tests

Significant heterogeneity was observed in some comparison models (P<0.10), and the results were shown in Table 2 and Table 3. We performed sensitivity analysis by removing each individual study sequentially for CTLA-4 60G/A (rs3087243) and CTLA-4 -1661A/G (rs4553808), respectively. The results indicated that the overall significance of the pooled ORs were not altered by any single study in the genetic models for the CTLA-4 60G/A (rs3087243) and CTLA-4 -1661A/G (rs4553808) polymorphism and cancer susceptibility, which suggest the stability and reliability of our overall results.

A Begg’s funnel plot and Egger’s test were used to assess the publication bias in our meta-analysis. The funnel plots were basically symmetric, and Egger’ test indicated no publication bias (P>0.05).

## Discussion

Recent findings in the field of tumor immunology have extended our understanding of interactions between immune system and tumor cells, it has become clear that the immune system can facilitate tumor progression through three phases: elimination, equilibrium, and escape [38], [39], [40]. CTLA-4 is a negative regulator of T-cell proliferation and activation, recent studies shows that it plays an important role in cancer immunosurveillance and may be involved in tumor development and progression [41]. It has been suggested that during early stages of tumorigenesis, CTLA-4 may elevate the T-cell activation threshold, attenuating the antitumor response and increasing cancer susceptibility [34]. However, studies focusing on the association of the CTLA-4 gene polymorphism with cancer susceptibility had controversial conclusions. The lack of concordance across these studies reflects limitation in the individual study, such as small sample sizes, ethnic difference and environment. Meta-analysis is a powerful tool which can overcome the problem of small sample size and inadequate statistical power of genetic studies of complex traits, summarize the results from different eligible studies and provide more reliable results than a single case-control study.

In this meta-analysis, we investigated the association between CTLA-4 60G/A (rs3087243) and CTLA-4 -1661A/G (rs4553808) and cancer risk. The subgroup analysis stratified by ethnicity, source of controls and cancer types were also performed. For CTLA-4 60G/A polymorphism, a total of 18 studies, comprising 5571 cases and 5567 controls, our meta-analysis on the available studies suggested no significant increased cancer risk in all of the genetic comparison models. The results were robust, which did not vary materially after we excluded the study with controls not in HWE. When we performed subgroup analysis by cancer type, we found the CTLA-4 60G/A (rs3087243) polymorphism is correlated to significant increased skin cancer. It was reported that CTLA-4 gene palys an important role in UV-induced immune suppression as well as in development of skin cancer, transgenic mice that express a skin-specific CTLA-4 antagonist, developed fewer skin tumors after chronic exposure to UV [42]. The CTLA-4 60G/A polymorphism is a key susceptibility locus for autoimmune and cancer, previous results indicated that presence of G alleles in polymorphic sites 60G/A polymorphism was associated with lower levels of membrane and cytoplasmic CTLA-4 in CD4+ T lymphocytes [43]. Moreover, in the subgroup analysis of source of controls, hospital-based group showed significant increased risk of cancers, and the results did not vary materially after we performed the sensitivity analysis. The remaining pooled ORs from this analysis were insignificant (all P>0.05).

For CTLA-4 -1661A/G (rs4553808) polymorphism, the SNP -1661A/G is located in the promoter region of CTLA-4. Allelic variants located in the promoter region may change the motif of functional DNA binding sites and then affect the affinities for the relevant transcription factors, which is important for regulation of transcription and alternative splicing. Previous data demonstrated that transcription factor c/EBP/β could bind to the -1661 sites in the presence of G allele, thereby regulate the function of CTLA-4 [29]. In our meta-analysis, we found significant association between CTLA-4 -1661A/G polymorphism and increased cancer risk in heterozygote model, dominant model and allele model. The results were very robust, which did not vary materially when we performed the sensitivity analysis (exclusion the study with controls not in HWE). In the subgroup analysis by ethnicity, we observed a significant association between increased cancer risk and Asian population, the sensitivity analysis by deleting studies with controls deviating from HWE still showed a significant association, which demonstrated our results were reliable. However, we did not found any significant increased cancer risk in Caucasians, ethnicity may be an essential biological factor which influences CTLA-4 -1661A/G polymorphism through gene to gene interaction. Moreover, when the data were stratified by cancer type, a significant increased cancer risk was observed in gastric cancer, breast cancer and other cancers. However, after we performed the sensitivity analysis, we did not found significant association between increased cancer risk and other cancers. In the subgroup analysis by source of controls, we found significant association between increased cancer risk and population based group. The remaining pooled ORs from this analysis were insignificant. Recent studies reported that CTLA-4 blockade could enhance the effect of a potent p53-expressing MVA vaccine, enhance the CTL response to p53 [44], [45]. These results suggest that the CTLA-4 -1661A/G polymorphism may be affect the expression and function of p53, and may be related to the tumor development.

To a certain extent, our meta-analysis still includes some limitations, which should be interpreted and taken into consideration. First, the lack of observations concerning gene-gene and gene-environment interactions could influence our results. Second, although the total number of studies was not small, there were still not sufficient eligible studies for us to analyse different types of cancers, such as breast cancer, renal cell carcinoma or lung cancer, more studies are needed to research the potential relationship between the CTLA-4 60G/A (rs3087243) and CTLA-4 -1661A/G (rs4553808) polymorphisms and cancer susceptibility. Third, the lack of detailed original data, such as the age and sex of the populations, smoking status, or alcohol consumption in the eligible studies may influence our further analyses. However, our meta-analysis also has many advantages. First, we searched all possible publications, and the total number of eligible studies was much larger than other previously published meta-analyses; therefore, our results are more convincing. Second, no publication bias was detected in our meta-analysis. Finally, the genotype distribution of controls did not agree with the HWE in the studies were excluded by sensitivity analysis, we revealed these studies did not affect the pooled ORs, so, our results were robust and reliable.

## Conclusions

In the present study, our meta-analysis suggests that the CTLA-4 -1661A/G polymorphism is a potential factor for the susceptibility of cancer, especially in gastric cancer, breast cancer and other cancers, and the CTLA-4 60G/A polymorphism is significantly associated with increased skin cancer risk. The effect of the CTLA-4 -1661A/G polymorphism on cancer especially exists in Asians and population based subjects. Due to existing limitations, additional well-designed studies with large sample size concerning gene-gene and gene-environment interactions are required to present more robust evidence for the association, and further molecular studies are warranted to clarify the effects of CTLA-4 60G/A and CTLA-4 -1661A/G polymorphisms on the susceptibility and progression of cancers.

## Supporting Information

Checklist S1 **PRISMA checklist.**
(DOC)
